# Visual management of medical things with an advanced color-change RFID tag

**DOI:** 10.1038/s41598-021-02501-x

**Published:** 2021-11-26

**Authors:** Ran Sun, Budi Rahmadya, Fangyuan Kong, Shigeki Takeda

**Affiliations:** 1 College of Engineering, Ibaraki University, Ibaraki, 316-8511 Japan; 2 Department of Computer Engineering, Faculty of Information and Technology, Andalas University, Limau Manis, Padang, Sumatera Barat 25175 Indonesia

**Keywords:** Engineering, Electrical and electronic engineering, Medical research, Health care

## Abstract

This paper proposes a visual management scheme of medical things with a color-change radio frequency identification (RFID) tag. The color-change RFID tag employs a specific RFID tag integrated circuit (IC) and a laminated pH-indicating paper. The IC has energy harvesting and switched ground functions, which enable it to generate electricity to the laminated pH-indicating paper. This phenomenon causes electrolysis of NaCl solution absorbed in the laminated pH-indicating paper. Electrolysis generates alkaline matter to change the color of the pH-indicating paper. This paper gives a new and sensitive structure of the laminated pH-indicating paper. The proposed advanced color-change RFID tag with new laminated pH-indicating paper succeeds in changing its color noticeably at a 1 m distance using an RFID reader radiating 1 W radio waves. The color change was observed 3–5 s after starting radio wave irradiation. The results of this experiment also confirm that the changed color can be held for over 24 h. Furthermore, two demonstrations of the visual management system of medical things (patient clothes and sanitizers) are presented.

## Introduction

The Internet of Medical Things (IoMT) can be defined as a specific type of the Internet of Things (IoT) fundamentals, principles, tools, techniques, and concepts that apply to the medical and healthcare sectors and domains^1^. Various studies of IoMT have been carried out, such as a remote health check-up^2^, disease detection^3^, and drug management^4^. Especially in today’s COVID-19 pandemic environment, the role of IoMT has become an important topic. IoMT enables doctors to monitor patients remotely and collect more data on patient health. The IoMT care systems support doctors’ check-ups and even detect some diseases automatically by artificial intelligence (AI)^5–7^. These also derive many extended issues, including real-time medical data sharing^8–10^, data processing^11,12^, and data security^13–15^. IoMT also helps medicine and drug management. With the help of IoT devices, patients obtain health data. These data help them manage medicines and drugs well^16^.

In the COVID-19 pandemic situation now, to avoid virus infection, the treatment of medical things, for example, the disposal and disinfection of protecting wears, face shields, masks, and gloves, and the management of medicines and vaccines, should be accurate and quick. Thus, a visual management scheme of medical things is required. In particular, some medical things are untouchable, such as rental patient clothes and vaccines. Remote controls are also required. As an IoT technique for remote item management, a radio frequency identification (RFID) technology is a good candidate^17,18^. It is low cost, remote and easy to combine sensors such as temperature sensing^19^ and vibration sensing^20^. RFID systems are divided into chip and chipless RFID systems. Both systems enable low-cost, wireless and passive unique identifications for medical things. These RFID systems are also easy to combine with various kinds of sensors. Many studies on chip and chipless RFID sensors have been carried out^19–25^. Chip RFID systems have been introduced to temperature^19^, vibration^20^, and moisture^21^ sensors, while chipless RFID systems have been applied to pH sensing^22^. Both chip and chipless RFID systems have been introduced to strain, crack, and corrosion sensors^23,24^. In addition, surface acoustic wave (SAW)-based chipless RFID systems have also been proposed, and they have been introduced to current sensing^25^. Measuring the above physical quantities is also useful for medical things’ management. However, since our research objective is to visualize the status of medical things, an RFID chip containing energy harvesting and switch ground functions is chosen.

**Figure 1:**
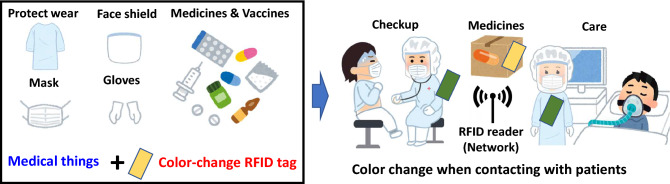
Visual management of medical things using a color-change RFID tag.

Our study focuses on the visual management of medical things using RFID tags. In this paper, we propose a visual management scheme with RFID tags as Fig. 1. The system is designed for inventory management and fast treatment of medical things, especially after contact with patients, such as the disposal or disinfection of protective wear, face shields, masks and gloves, and the remote inventory management of medicines and vaccines. To discriminate the specific things, a staff member using an ordinary RFID system needs to read them one by one by adjusting radiation power values if needed. On the other hand, in our proposed system, an RFID reader sends commands to enable visual management for all the tag populations at once. With this visualization, the colors of the specific RFID tags can be changed, and a staff member can easily identify the specific things.

In the proposed visual management scheme, the color-change RFID tags^26^, which can change color under the interrogation by an RFID reader, are attached to the medical things. The RFID reader is installed in places such as medicine storerooms, check-up rooms, and sickrooms as Fig. 1. Using the proposed management system, we can change the color of the specific RFID tag as a sign of disposal, disinfection, or other treatment. Thus, visual management is available. The color-change function is realized by the pH-indicating paper’s color change caused by the electrolysis of NaCl (salt) solution. We have investigated a preliminary color-change RFID tag using an anthocyanin solution as a pH-indicator^26^. However, the color change was slow and the area was narrow^26^. The slow color-change speed is because of the characteristics of an anthocyanin solution. Thus, we need to find a better type of pH indicator with a fast color-change reaction. Moreover, the electrode structure also affects the color-change speed and narrows the size of the visible area. In a previous study^26^, overlapping electrodes (cathode and anode electrodes) were chosen to obtain a strong static electric field distribution. However, since the cathode and anode generate $\mathrm{H}_2$ and $\mathrm{Cl}_2$, respectively, if these electrodes are too close together, they generate the acid HCl, causing a neutralization reaction. If they are too far apart, the electrolysis that causes a color change will be slow, and the area will be narrow because the static electric field distribution is weakened. Hence, the cathode and anode need to maintain a suitable distance and be constructed appropriately.

In this paper, to solve the above problems, we enhance the structure of the laminated pH-indicating paper. To this end, we introduce a new electrode structure and adopt a better type of pH-indicating paper. To confirm the practicalities of the proposed system, we conduct some demonstrations for medical things management (e.g., patient clothes and sanitizers, similar to medicines and vaccines). Moreover, we analyze the color-changing performances (color-changing distance and speed) and color-holding performances (color retention time) of the advanced color-change RFID tag. In these analyses, detailed comparisons of the color-changing and color-holding performance of different pH indicators are also presented. Since $\mathrm{CO}_2$ in air causes a neutralization reaction, transparent films constituting a laminated pH-indicating paper should be joined in an appropriate way to prevent contamination by $\mathrm{CO}_2$. The performance of this laminated structure is also investigated.

## Results and discussions

We demonstrate two IoMT applications: one is for managing patient clothes, and the other is for managing sanitizers.

### Patient clothes management

**Figure 2:**
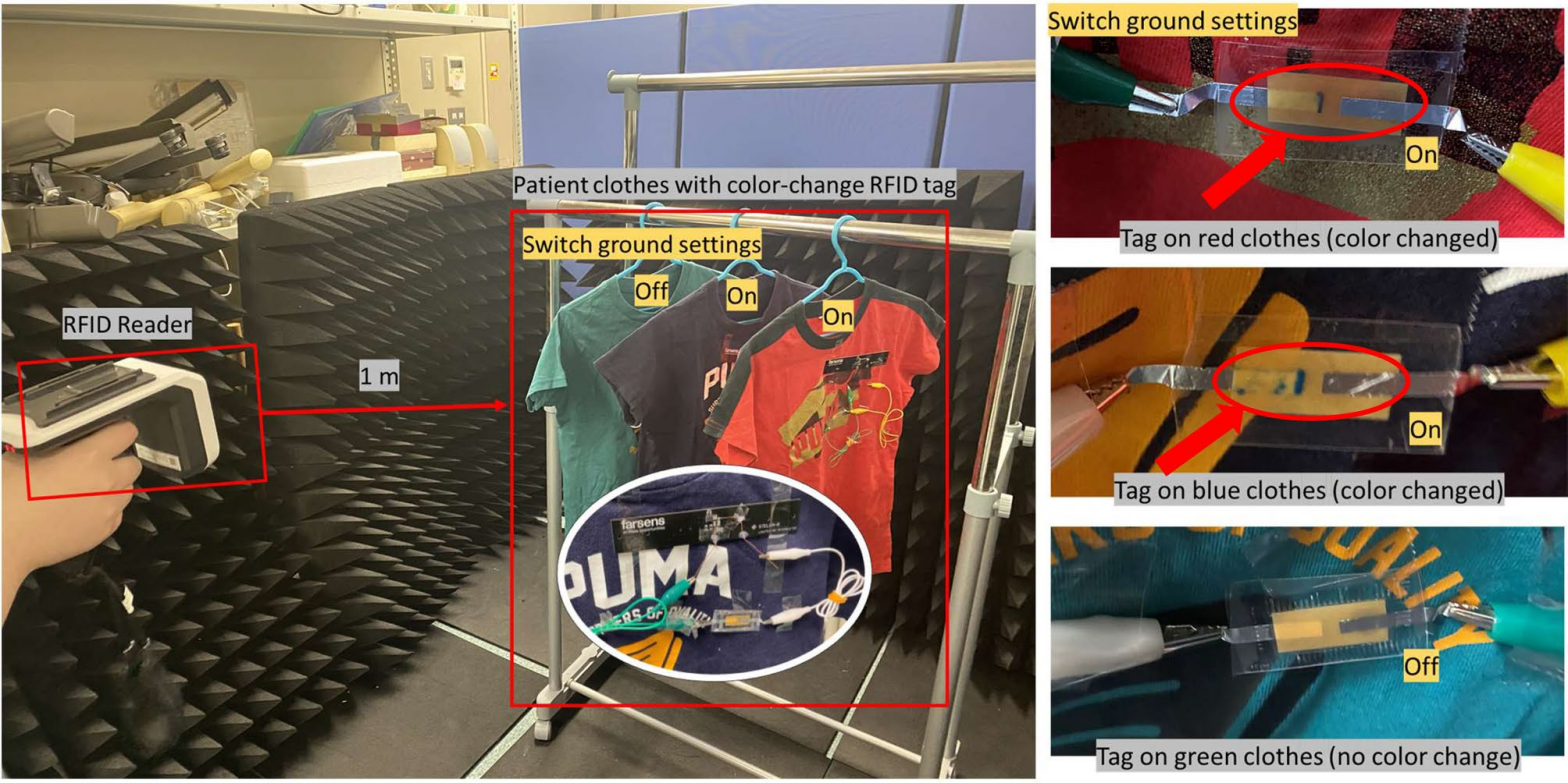
Demonstration of IoMT application for managing patient clothes after 30 s of management (RFID reader irradiation). The red and blue clothes are set as the target clothes (Switch ground setting = ‘on’).The color-change positions are specified by red arrows and circles.

Figure 2 shows the demonstration and its results of the IoMT application for managing patient clothes. The proposed color-change RFID tag is installed on the clothes, and the RFID reader is set at a distance of approximately 1 m. In this demonstration, the red and blue clothes are set as the target clothes (Switch ground setting = ‘on’). The switch ground is a built-in function of the RFID integrated circuit (IC) we employed, which is explained in the later part.

We turn on the RFID reader irradiation by a Bluetooth-connected computer during the 30 s’ management of patient clothes. As a result, the proposed system succeeds in changing the tags’ colors of the target clothes. The right part of Fig. 2 shows photographs of the color-changed RFID tag after 30 s of management (irradiation). We can see that the tags of the red and blue clothes change their colors, and there is no color change on the tag of the green clothes.

### Sanitizer management

**Figure 3:**
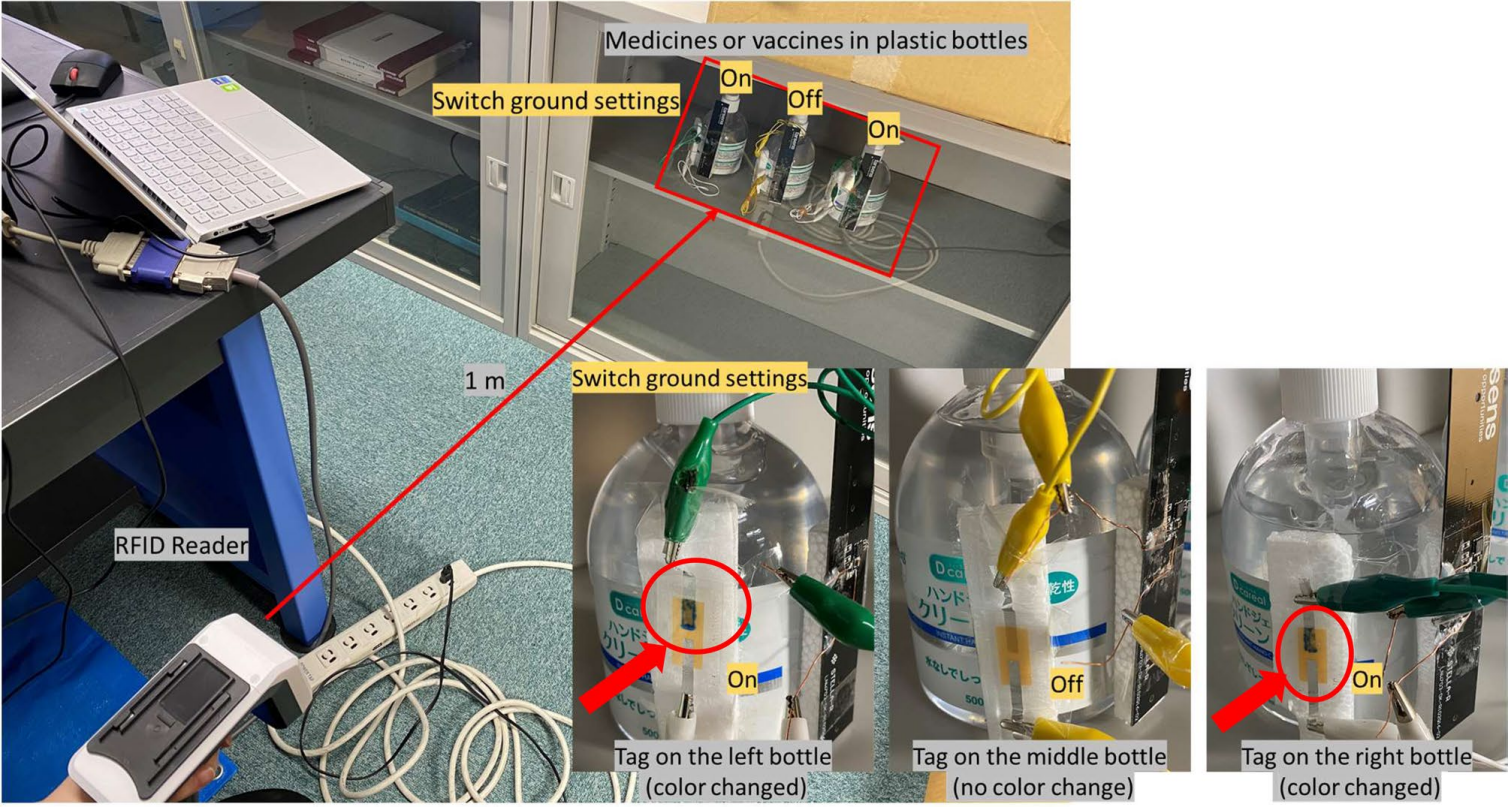
Demonstration of an IoMT application for managing sanitizers in plastic bottles after 30 s of management (RFID reader irradiation). The left and right sanitizers are set as the targets (Switch ground setting = ‘on’). The color-change positions are specified by red arrows and circles.

Figure 3 shows a demonstration and its result of the IoMT application for managing sanitizers. Sanitizers can also be considered substitutes for medicines or vaccines. The proposed color-change RFID tag is installed on the bottles of sanitizers, and the RFID reader is also set at a distance of approximately 1 m. Note that the sanitizers are in the locker, which means that we should manage the sanitizers through the glass. We set the left and right sanitizers as the targets.

We can see that the tags on the left and right sanitizers change their colors after 30 s of management (irradiation), and there is no color change on the tag of the middle sanitizer as Fig. 3.

## Method

In this section, we introduce the framework of the proposed color-change RFID tag.

**Figure 4:**
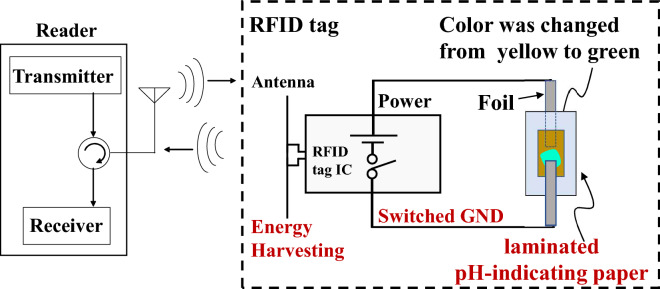
Framework of color-change RFID tag.

Figure 4 illustrates the framework of the proposed color-change RFID tag^26^. The color-change RFID tag consists of an RFID tag integrated circuit (IC) with energy harvesting and switch ground functions and a piece of laminated pH-indicating paper that has absorbed the NaCl (salt) solution.

**Figure 5:**
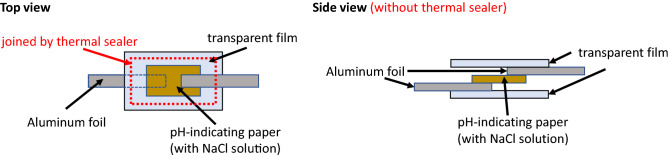
New structure of laminated pH-indicating paper.

Figure 5 depicts the new structure of the laminated pH-indicating paper. The laminated pH-indicating paper was prepared by the following procedure. The pH-indicating paper first absorbs a NaCl solution (0.18 g/ml NaCl concentration), and then the pH-indicating paper, aluminum foil and transparent film are laminated, as shown in Fig. 5. The pH-indicating paper is sandwiched by aluminum foil electrodes and laminated with transparent plastic films. Aluminum does not rust when it is exposed to a NaCl solution for a long time. Aluminum is also widely used to fabricate RFID tag antennas. Sufficient electrolysis phenomena needed to change the color of the pH-indicating paper were experimentally confirmed using aluminum electrodes. Therefore, electrodes made from aluminum are used in this paper. If there are better and less expensive materials, they can also be adopted. The transparent films are finally joined together by a thermal sealer to prevent air contamination ($\mathrm{CO}_2$) and drying of the solution.

Under the interrogation by the RFID reader, the energy harvesting IC generates electricity. This causes the electrolysis of the NaCl solution absorbed in the laminated pH-indicating paper. With electrolysis, the cathode generates alkaline matter ($\mathrm{OH}^-$) so that the color of the pH-indicating paper changes. The product number of the pH-indicating paper used in this paper is Q/GHSC 1571–2009, which changes its color to green (9.0 < pH < 10.0) or blue (pH> 10.0) from yellow when pH > 8.0. The Q/GHSC 1571–2009 pH-indicating paper is a commercially available yellow paper. This yellow pH-indicating paper is based on a thymolsulfonephthalein (thymol blue) acid-base indicator, which has an initial yellow color and changes to blue in alkaline substances. However, two reasons cause the neutralization reaction, which makes the changed color fade. One reason is the $\mathrm{CO}_2$ in the air. Thus, we use the laminated structure to avoid $\mathrm{CO}_2$. The other is the acid (HCl) from the reaction between $\mathrm{H}_2$ and $\mathrm{Cl}_2$, which are generated in the cathode and anode, respectively. Therefore, the cathode and anode need to maintain a suitable distance. If the distance is too long, the electrolysis (color change) would be slow because the static electric field becomes weak. In this paper, we set a distance of 3 mm as a suitable distance.

**Figure 6:**
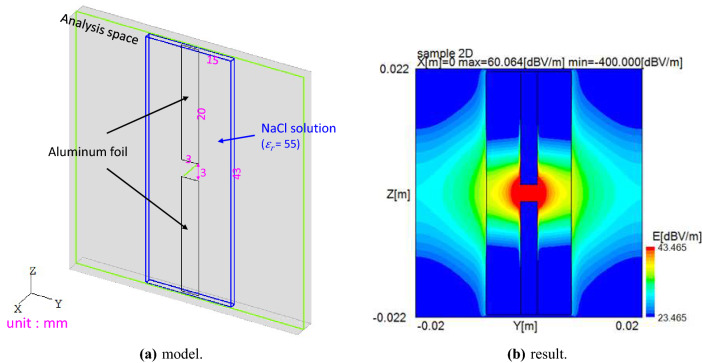
Static electric field analysis of the electrodes with a laminated pH-indicating paper part (applied voltage = 1 V). (**a**) model. (**b**) result.

A static electric field of the laminated pH-indicating paper part is analyzed to confirm its electrolysis performance. Figure 6a shows the static electric field analysis model of the electrodes of the laminated pH-indicating paper part. Figure 6b presents the static electric field analysis result of the electrodes of the laminated pH-indicating paper part. The static electric field analysis result is computed by the static electric field analysis software OpenSTF^27^ based on solving LaPlace’s equations. We can see that the static electric field is concentrated at the edge of the electrodes.

**Figure 7:**
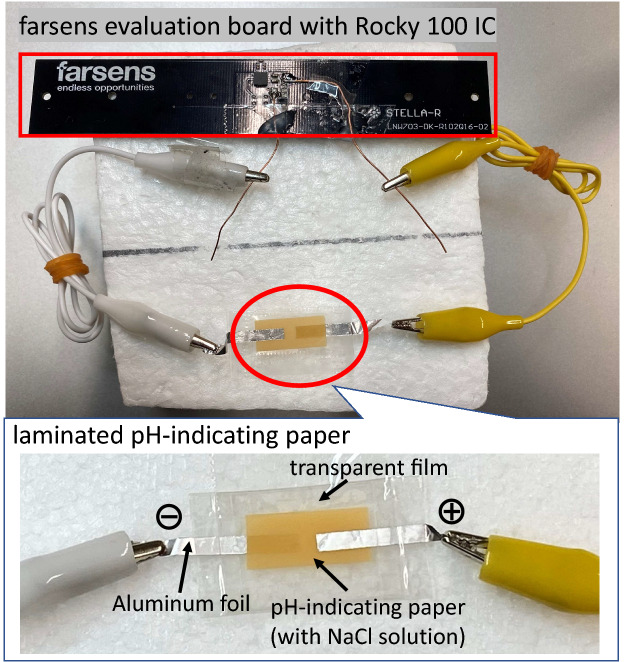
Fabricated prototype sample of color-change RFID tag that exploited an evaluation board (Stella-R)^28^ of a Rocky 100 IC (Farsens)^29^.

Figure 7 shows the fabricated prototype sample of the color-change RFID tag, which is based on a Stella-R evaluation board^28^ with a Rocky 100 IC (Farsens)^29^. The Rocky 100 IC has an energy harvesting function and switch ground function. The switch ground of the IC can be set as ‘on’ or ‘off’ to switch the energy harvesting function, and each tag can be specified by the electronic product codes (EPCs). Thus, it is possible remotely to change the colors of the target tags. Note that harvested energy is not applied to the laminated pH-indicating paper, and the RFID tag IC does not change its color when the switch ground is set to the ‘off’ state. This is because the RFID IC, Rocky 100, has a switch ground function that switches the energy harvesting status from ‘off’ to ‘on’ and vice versa. This switch ground function can also prevent color changes caused by unintended exposure to radio waves. The evaluation board with the Rocky 100 IC has a default color-change function using light-emitting diode (LED) lighting. However, LED lighting requires much higher power than our proposed color-change scheme, and the LED can emit light only when the RFID reader irradiates the radio waves. Conversely, the proposed color-change tag can hold the changed color even after the irradiation of the radio waves by the RFID reader.

## Experimental basic performance analysises

In this section, we confirm the characteristics of the proposed color-change RFID tag. Both the color-changing performance and the color-holding performance are investigated.

### Color-changing performance

To evaluate the color-changing performance, two metrics are used: the color-changing speed and the available distance.

**Figure 8:**
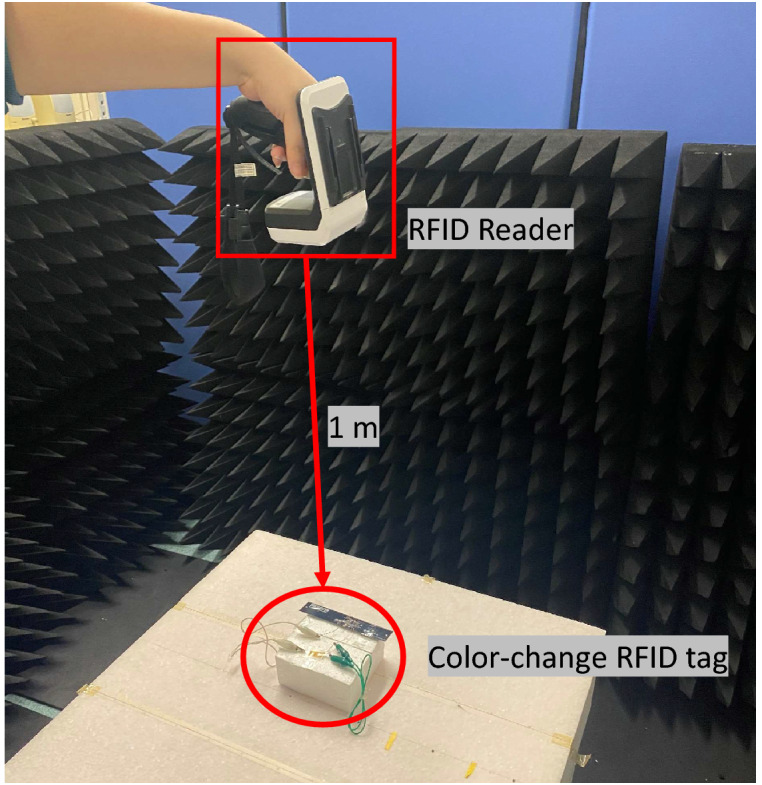
Irradiation experiment of color-change RFID tag at a 1 m distance using the RFID reader (DOTR-3200^30^) irradiating 1 W radio waves.

Figure 8 shows the experimental environment of the RFID tag at a 1 m distance using the RFID reader irradiating radio waves. An RFID reader with a circularly polarized antenna, DOTR-3200^30^, which has a transmission power of 1 W, was used. The radio wave irradiation time and frequency channel were set as 400 ms and 920.4 MHz, respectively. As for comparison, the pH-indicating paper using anthocyanin or phenolphthalein was also evaluated in the experiment. The color-changing performances are summarized in Table 1.

**Table 1:** Color-changing performances of the color-change RFID tag using Q/GHSC 1571–2009, phenolphthalein and anthocyanin pH-indicating paper.

pH-indicating paper	Distance	Color-changing speed
Q/GHSC 1571–2009	Over	3–5 s
Phenolphthalein	1 m	(Noticeable color-change in 30 s)
Anthocyanin^26^	0.5 m	60 s

**Figure 9:**
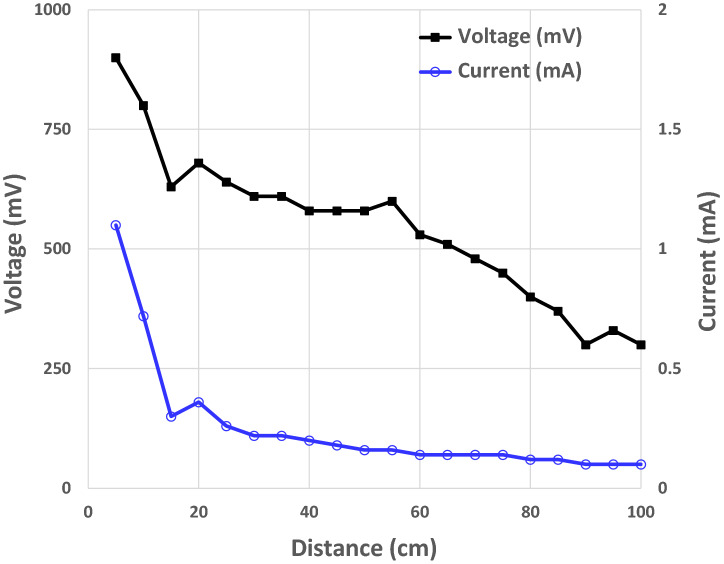
Voltage and current versus irradiation distance on the proposed color-change RFID tag.

At the color-changing speed, both the color-change RFID tag with the proposed Q/GHSC 1571–2009 pH-indicating paper and the tag with the phenolphthalein pH-indicating paper start color-change at the edge of the cathode foil in 3–5 s under irradiation at a 1 m distance. After 30 s of irradiation, we can see a noticeable color change around the cathode foil. The color-changing performance is sufficient for practical use. The tag’s color-changing performance with the anthocyanin pH-indicating paper is serious, and the anthocyanin pH-indicating paper starts to change color in 60 s under irradiation at a 0.5 m shorter distance. Moreover, the result that the color change begins from the edge of the cathode foil follows the static electric field analysis in Fig. 6b. The voltage and current on the proposed laminated pH-indicating paper under irradiation at different distances are shown in Fig. 9. The power and switch ground terminals of the RFID IC are fed directly to the laminated pH-indicating paper, as shown in Fig. 4. To measure the voltage and current applied to the laminated pH-indicating paper, voltage and current meters were used. The current meter was inserted in series with the power terminal, and the voltage meter was inserted in parallel with the electrodes of the laminated pH-indicating paper to measure the currents and voltages applied to the laminated pH-indicating paper. A shorter irradiation distance results in higher color-changing performance. We confirmed that the maximum available range of the proposed color-change RFID tag is over 1 m. The power consumption is approximately 30 $\mu$W at 1 m, which can be calculated from Fig. 9.

### Color-holding performance

**Figure 10:**
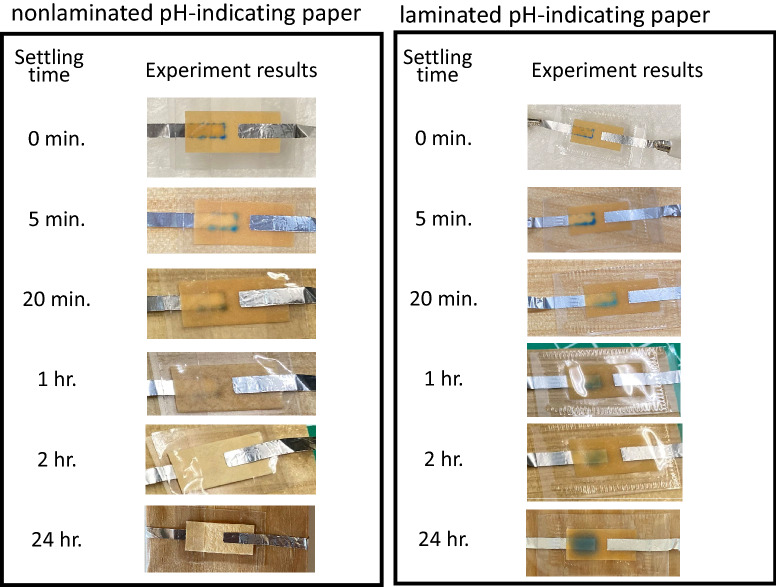
Color retention time of the proposed color-change RFID tag with the nonlaminated/laminated Q/GHSC 1571–2009 pH-indicating paper.

**Figure 11:**
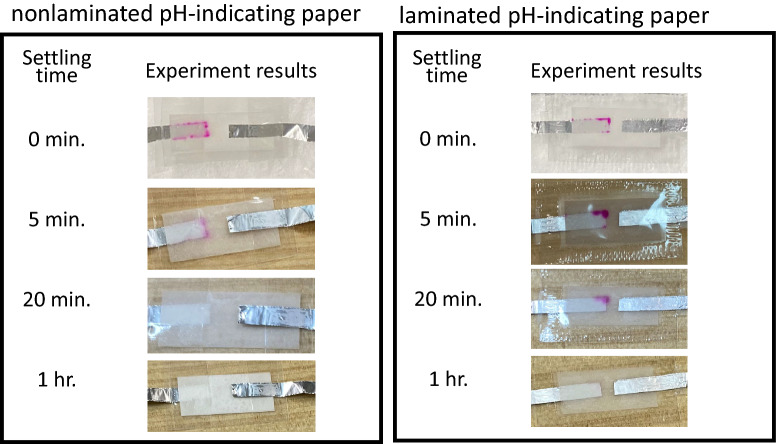
Color retention time of the proposed color-change RFID tag with the nonlaminated/laminated phenolphthalein pH-indicating paper.

**Figure 12:**
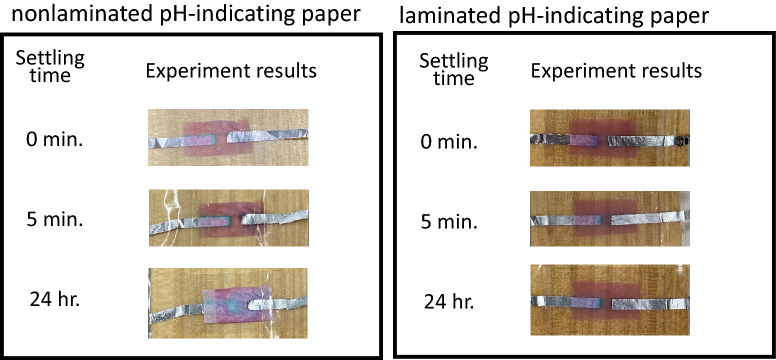
Color retention time of the proposed color-change RFID tag with the nonlaminated/laminated anthocyanin pH-indicating paper.

For the color-holding performance, we check the color retention time. Figures 10, 11 and 12 show the color retention time of the proposed color-change RFID tag with the nonlaminated/laminated Q/GHSC 1571–2009, phenolphthalein and anthocyanin pH-indicating paper, respectively. We can see that the nonlaminated tags become dry in 2 h, and the colors of the tags using Q/GHSC 1571–2009 and phenolphthalein pH-indicating paper disappear soon. With lamination, the color retention times become longer. The reason is the prevention of $\mathrm{CO}_2$ and drying of the solution.

As Fig. 10, the proposed laminated tag with the Q/GHSC 1571–2009 pH-indicating paper can hold the changed color for over 24 h. Although the color becomes faint as time goes on, the remaining color is enough to decide whether the medical things have been used for a certain period, even if 24 h have passed. In Fig. 11, since the color-changing is white to red, we can observe the changed color clearly. However, as an unstable material of phenolphthalein, the retention time of the tag is just 1 h, even with lamination. In Fig. 12, the changed color can remain even without lamination. However, its color-changing performance is low. In summary, the laminated color-change tag with the Q/GHSC 1571–2009 pH-indicating paper has the best performance overall. The color-holding performance is concluded in Table 2.

**Table 2:** Color-holding performances (color retention time) of the color-change RFID tag using the Q/GHSC 1571–2009, phenolphthalein and anthocyanin pH-indicating paper.

pH-indicating paper	Color retention time	Color retention time
	(Nonlaminated)	(Laminated)
Q/GHSC 1571–2009	1 h	Over 24 h
Phenolphthalein	20 min.	1 h
Anthocyanin^26^	Over 24 h	Over 24 h

## Conclusion

In this paper, we proposed a visual management system of medical things with a color-change RFID tag that adopts a new structure of laminated pH-indicating paper. In the new structure, we introduced a new electrode structure and adopted a better type of pH-indicating paper. We fabricated a prototype of the color-change RFID tag. We succeeded in demonstrating an IoMT application for managing patient clothes and sanitizers to show the effectiveness of the proposed visual management system.

Moreover, we carried out a basic irradiation experiment of the color-change RFID tag at a 1 m distance using an RFID reader radiating 1 W radio waves. As results, the proposed RFID tag starts color change after 5 s of irradiation and changes to a noticeable color after 30 s of irradiation. Moreover, the RFID tag can hold the changed color for over 24 h. For future works, we investigate other electrolytes instead of the NaCl solution to avoid the color fade caused by the acid.
